# Phenotypic adaptations of *Leishmania donovani* to recurrent miltefosine exposure and impact on sand fly infection

**DOI:** 10.1186/s13071-020-3972-z

**Published:** 2020-02-22

**Authors:** Sarah Hendrickx, Lieselotte Van Bockstal, Dimitri Bulté, Annelies Mondelaers, Hamide Aslan, Luis Rivas, Louis Maes, Guy Caljon

**Affiliations:** 1 Laboratory of Microbiology, Parasitology and Hygiene (LMPH), University of Antwerp, Antwerp, Belgium; 2 Centro de investigaciones Biológicas - CSIC, Madrid, Spain

**Keywords:** Miltefosine, *Leishmania*, Fitness, *Lutzomyia longipalpis*, Drug uptake

## Abstract

**Background:**

Since the introduction of miltefosine (MIL) as first-line therapy in the kala-azar elimination programme in the Indian subcontinent, treatment failure rates have been increasing. Since parasite infectivity and virulence may become altered upon treatment relapse, this laboratory study assessed the phenotypic effects of repeated *in vitro* and *in vivo* MIL exposure.

**Methods:**

Syngeneic *Leishmania donovani* lines either or not exposed to MIL were compared for drug susceptibility, rate of promastigote multiplication and metacyclogenesis, macrophage infectivity and behaviour in the sand fly vector, *Lutzomyia longipalpis*.

**Results:**

Promastigotes of both *in vitro* and *in vivo* MIL-selected strains displayed a slightly reduced drug susceptibility that was associated with a reduced MIL-accumulation linked to a lower copy number (disomic state) of chromosome 13 harboring the *miltefosine transporter* (*LdMT*) gene. *In vitro* selected promastigotes showed a lower rate of metacyclogenesis whereas the *in vivo* derived promastigotes displayed a moderately increased growth rate. Repeated MIL exposure did neither influence the parasite load nor metacyclogenesis in the sand fly vector.

**Conclusions:**

Recurrent *in vitro* and *in vivo* MIL exposure evokes a number of very subtle phenotypic and genotypic changes which could make promastigotes less susceptible to MIL without attaining full resistance. These changes did not significantly impact on infection in the sand fly vector.
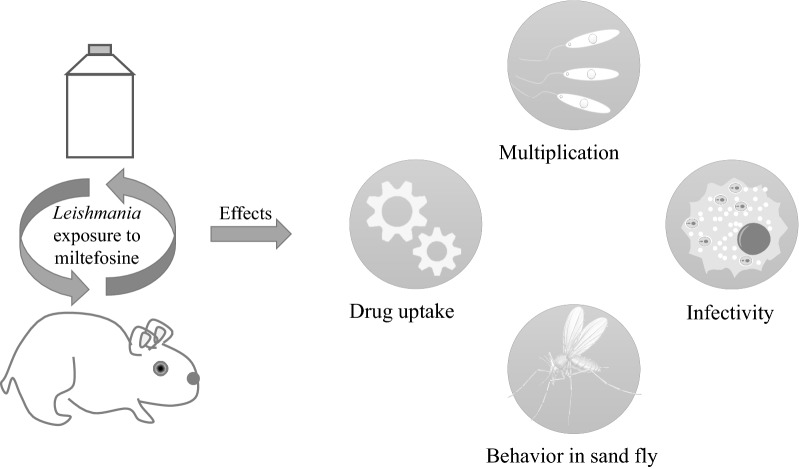

## Background

Although miltefosine (MIL) monotherapy was advocated about one decade ago as first-line treatment against visceral leishmaniasis (VL) in frame of the kala-azar elimination programme in the Indian subcontinent, its use has now largely been abandoned owing to the increase in therapeutic failures [1]. However, evidence for the involvement of drug resistance has remained scarce and the few reports of MIL resistance in clinical isolates were published only very recently [2]. The multifactorial causes of treatment failure are still not fully understood and involvement of the parasite’s (epi-) phenotype has been suggested [3, 4]. Clinical isolates from relapse patients revealed alterations in parasite fitness rather than in drug susceptibility [3, 5]; however, the lack of large sets of paired pre- and post-treatment samples precludes direct comparison of syngeneic strains. Most laboratory studies circumvented this problem by repeatedly exposing promastigotes to MIL, hence generating resistant strains retaining an identical genetic background to the parent wild-type (WT). This way, refractoriness to MIL could be related to active drug efflux *via* LMDR1/LABCB4 or LABCG6 that are both P-glycoprotein-like transporters from the *Leishmania* ABC (ATP-binding cassette) family [6–9]. Later on, MIL resistance was linked to mutations in the putative *L. donovani* MIL transporter (LdMT) and/or its β-subunit LdRos3, leading to a defective inward drug transport [10–12]. Although the broader impact of these mutations is still a topic of debate and may even be species-related [13, 14], MIL resistance definitely appears to be associated with alterations in parasite fitness. Although selection on promastigotes fairly rapidly resulted in resistance, repeated exposure of intracellular amastigotes both *in vitro* and *in vivo* did not result in reduced MIL susceptibility [15, 16]. This may actually reflect the situation in the field where parasites recovered from patients retain an unaltered MIL susceptibility profile.

The present laboratory study used intracellular amastigotes of field isolates from Nepal that were repetitively exposed to MIL, both *in vitro* and *in vivo* in the hamster model. The phenotypic characteristics of the WT parent strain and derived MIL-exposed lines were comparatively evaluated to explore their impact on drug responsiveness, *in vitro* growth characteristics and transmission potential by the sand fly vector.

## Methods

### Animals

Female Swiss mice (20–25 g) and female golden hamsters (80–100 g) were obtained from Janvier (Le Genest-Saint-Isle, France) and kept in quarantine for at least 5 days before infection. Food for laboratory rodents (Carfil, Arendonk, Belgium) and drinking water were available *ad libitum*. Hamsters were allocated to experimental units of 2 animals each.

### *Leishmania* parasites

Intracellular amastigotes of the antimony (Sb)-resistant *L. donovani* clone MHOM/NP/03/BPK275/0cl18 were exposed *in vitro* to five successive treatment rounds of MIL (= BPK275/0cl18 MIL) [15]. Because of difficulties for its *in vivo* adaptation, another clonal line (= MHOM/NP/02/BPK282/0cl4) showing better infectivity in hamsters was used for the *in vivo* resistance selection experiments, as described elsewhere [16]. In brief, infection inoculates containing 2 × 10^7^ spleen-derived amastigotes in phosphate-buffered saline (PBS) were administered by intracardial injection under isoflurane inhalation anesthesia. The general condition and body weight of the animals were monitored daily to evaluate the course of infection. Three weeks after infection, the animals were treated orally with MIL (20 mg/ml in PBS) for 5 days at 40 mg/kg single injection dose (s.i.d.) and followed-up until clinical signs of relapse were noted (decrease in body weight, poor general appearance), hence enabling successive treatment-relapse cycles [16]. After five cycles, the resulting parasite population was harvested from the spleen and expanded *in vitro* as promastigotes (= BPK282/0cl4 MIL) in MIL-free medium. Both parent wild-type (WT) isolates had been obtained from bone-marrow aspirates of patients from the Terai endemic region in Nepal (BP Koirala Institute, Dharan, Nepal) within the frame of the EU-Kaladrug-R project and were provided by the Institute of Tropical Medicine (Antwerp, Belgium). Both strains were typed as *L. donovani* by cysteine proteinase B (CPB)-PCR-RFLP and their full genome sequences are available [17, 18]. Promastigotes were cultured in HOMEM medium (Life technologies, Ghent, Belgium) supplemented with 10% inactivated fetal calf serum (iFCS).

### Drug formulations and preparation

For the *in vitro* work, a stock solution of 20 mM MIL (MW = 407.57) (Carbosynth, Berkshire, UK) was prepared in PBS and stored at 4 °C. For the *in vitro* uptake studies, fluorescent BODIPY-MIL analogue 2 was used [19]. For the treatment of animals, MIL was formulated in distilled water at 20 mg/ml.

### Drug susceptibility determination

Standard promastigote and intracellular amastigote assays were performed to assess MIL susceptibility. Primary peritoneal macrophages were harvested from starch-stimulated female Swiss mice. About 48 h after seeding, the cells were infected with stationary-phase promastigotes at a multiplicity of infection of 20:1. Twenty-four hours later, residual extracellular promastigotes were removed by washing and 2-fold MIL dilutions were subsequently added to the plates. After another 5 days of incubation at 37 °C and 5% CO_2_, the plates were stained with Giemsa and 50% inhibitory concentrations (IC_50_) were determined microscopically. To evaluate promastigote susceptibility, early log-phase promastigotes were exposed to two-fold MIL dilutions for 72 h, after which IC_50_-values were determined upon addition of resazurin and fluorimetric reading [20].

### Evaluation of MIL uptake rates

Intracellular accumulation of BODIPY-MIL [19] was compared between promastigote stages of the WT parent and the MIL-selected strains. Early log-phase (96 h) and stationary-phase (168 h) promastigotes were suspended in RPMI-1640 medium without phenol red at a concentration of 5 × 10^6^ cells/ml and were labeled with 0.5 µM BODIPY-MIL in the dark for 1 h at room temperature. Upon incubation, the non-internalized drug was removed by washing the parasites twice with ice-cold PBS containing 1% bovine serum albumin, resuspended in RPMI-1640 medium and kept on ice to block outward drug transport. Quantification of intracellular MIL accumulation was achieved by measuring fluorescence intensity in the FL2-channel by flow cytometry (FACSCalibur^®^). TO-PRO®-3 iodide (Molecular Probes^®^, Eugene, OR, USA) was used for live/dead staining and 100,000 events per sample were recorded [21]. Non-treated parasites were included as negative control. Data analysis was performed using BD CellquestPro® software.

### Parasite fitness analysis

#### Promastigote growth

Promastigote growth curves were made [22] to compare the *in vitro* growth of the WT parent and MIL-exposed derived strains. After passage through fine needles (21 G × 1½″, 0.8 × 40 mm and 25 G × 5/8″, 0.5 × 16 mm) to break clustering, the promastigotes were diluted in PBS and counted by flow cytometry. Exactly 5 × 10^5^ log-phase promastigotes/ml were seeded in 5 ml HOMEM and their number was determined flow cytometrically (FACSCalibur^®^) every 24 h for a total of 10 days. Three independent repeats of each strain were run in parallel and growth curves were made by plotting the average parasite density ± the standard error of the mean (SE) over time.

#### Morphological evaluation of metacyclogenesis

To evaluate metacyclogenesis at each time point, the flagellum/cell body length ratio was determined. Promastigotes were air-dried on a glass microscopy slide, fixed with methanol, Giemsa-stained and analyzed with bright field microscopy (Axiovert 200m^®^; Carl Zeiss, Zaventem, Belgium) using Zeiss Axiocam MRm^®^. The flagellum and cell body lengths of at least 50 parasites per slide were determined using the Axiovision^®^ software and the percentage of metacyclics present in each sample was calculated. Parasites were considered metacyclic when the flagellum/cell body ratio exceeded 2 [22].

#### Promastigote infectivity

The *in vitro* infectivity of WT parent and MIL-exposed promastigotes was compared by infecting primary peritoneal mouse macrophages at a 20/1 parasite/macrophage ratio and subsequent microscopic assessment of the infection index (total number of amastigotes counted / total number of cells counted) upon Giemsa-staining 24 h after infection. Promastigote viability was determined flow cytometrically before infection with the single-stain viability dye TO-PRO^®^-3 iodide (10 µM) (Molecular Probes^®^) to ascertain identical infection inoculates for each strain, hereby ensuring full comparability. The percentage of infected cells was calculated to determine the cell infectivity.

#### Promastigote multiplication and metacyclogenesis in the sand fly vector

The effect of repeated MIL exposure on overall parasite fitness in the sand fly vector was evaluated in a *Lutzomyia longipalpis* colony maintained under standard conditions [23]. To simulate natural transmission, female sand flies (3–5 days-old) were fed with heat-inactivated mouse blood containing 5 × 10^6^ promastigotes/ml through a blood feeding device covered with a chicken-skin membrane. After 24 h, blood-fed females were isolated and further maintained at 26 °C. To follow-up the infection over time, the flies were dissected on 2, 5, 7 and 9 days post-infection (dpi) to microscopically check the presence and localization of promastigotes in their guts, or by crushing the total gut content in PBS and microscopically quantifying the infection intensity using a KOVA counting chamber. Metacyclogenesis was evaluated morphometrically by light microscopy in parallel. The experimental infections were performed with an independent repeat for each strain. Parasites were also isolated from the vector on 7 dpi and cultivated in HOMEM promastigote medium supplemented with 5% penicillin-streptomycin. When sufficiently dense log-phase cultures were obtained, post-fly promastigotes and intracellular amastigotes were used for routine MIL susceptibility determination, as described above.

### MIL transporter copy number comparison by qPCR

Given the known plasticity in chromosome copy number for chromosome 13 (harboring the *LdMT* MIL-transporter gene) and chromosome 32 (harboring the *LdROS3*-subunit gene), the copy number of both was determined by qPCR. Chromosomes with stable somy (and preferentially large size) were selected as references based on previous research, i.e. chromosome 24 (stably disomic), chromosome 31 (mostly tetrasomic) and chromosome 36 (stably disomic) [24].

Upon selection of reference genes based on gene lists for annotated chromosomes (https://www.ncbi.nlm.nih.gov/genome/?term=leishmania+donovani), the single copy nature of these genes was confirmed by BLAST in the TriTryp database and conservation of the gene sequence was checked in *L. donovani* (BPK282A1) and *L. infantum* (JPCM5). Primers were designed to amplify a 116-bp region in chromosome 24, a 64-bp region in chromosome 31 and a 96-bp region in chromosome 36 by using online software (http://www.ncbi.nlm.nih.gov/tools/primer-blast/) and were synthesized by Integrated DNA Technologies (IDT, Belgium). DNA extraction of promastigote cultures was performed using the QIAamp DNA Mini and Blood Mini Kit (Qiagen, Hilden, Germany) according to the manufacturer’s instructions. Resulting DNA concentrations and purities were estimated by NanoDrop2000c spectrophotometer (Thermo Fisher Scientific, Merelbeke, Belgium). The Step One Plus real-time PCR system (Applied Biosystems, Bleiswijk, the Netherlands) was used for all PCR assays and melt curve analyses. Each assay was run according to the conditions stated in Additional file 1: Table S1 in technical duplicate. Resulting Cq-values were used to determine the corresponding chromosome copy numbers. The normalized relative quantity of chromosome 13 (containing *LdMT)* and chromosome 32 (containing *LdROS3*) was calculated using Qbase software version 3.2 and using chromosomes 24, 31 and 36 as references.

### Statistical analysis

All statistical analyses were performed using Graphpad Prism version 6.00 software. Statistical differences between WT parent and MIL-exposed derived strains and between the different time points within one group were determined using 2-way ANOVA with Bonferroni *post-hoc* comparisons for parasite growth, parasite morphology and infection indices. Morphological and infection indices inter-group comparisons were carried out using the non-parametric Friedman test followed by Dunnʼs *post-hoc* comparisons. Statistical comparisons of relative copy numbers and sand fly parasite load and percentage of metacyclics were made using the Student’s t-test. Tests were considered statistically significant if *P* < 0.05.

## Results

### Drug susceptibility determination

Repeated MIL exposure of *L. donovani* amastigotes *in vitro* (BPK275/0-cl18 MIL) or *in vivo* (BPK282/0-cl4 MIL) did not result in a significant increase in intracellular amastigote IC_50_ in the *in vitro* drug susceptibility assay (Table 1). The marginal decrease in promastigote susceptibility following both *in vitro* and *in vivo* selection was insufficient to account for full MIL resistance [25]. To evaluate the stability of this ‘altered’ promastigote phenotype upon passage in the sand fly, MIL susceptibility was determined on ‘post-fly’ derived promastigotes with negligible differences in susceptibility between the different ‘post-fly’ subcultures. After passage in the sand fly, MIL IC_50_ values show an overall trend to be lower in both promastigotes and amastigotes (Table 1).

**Table 1:** Drug susceptibility of the wild-type (WT) parent and the derived isolates exposed for five cycles to MIL

Strain	Intracellular amastigote IC_50_ ± SE (µM)	Post-fly amastigote IC_50_ ± SE (µM)	Promastigote IC_50_ ± SE (µM)	Post-fly promastigote IC_50_ ± SE (µM)
BPK275/0-cl18 WT	2.4 ± 0.3	0.4 ± 0.1	5.8 ± 1.0	2.8 ± 0.3
BPK275/0-cl18 MIL	3.1 ± 0.4	1.4 ± 0.1	9.8 ± 1.2	4.5 ± 0.4
BPK282/0-cl4 WT	2.4 ± 0.4	0.6 ± 0.1	2.6 ± 0.8	5.0 ± 0.6
BPK282/0-cl4 MIL	1.2 ± 0.1	1.0 ± 0.2	9.7 ± 2.5	7.2 ± 1.3

*Notes*: Susceptibility of the different strains is expressed as the average 50% inhibitory concentration (IC_50_) ± standard error of the mean (SE) and is the result of at least four independent experiments run in duplicate

### MIL transporter gene copy number determination by qPCR

**Fig. 1:**
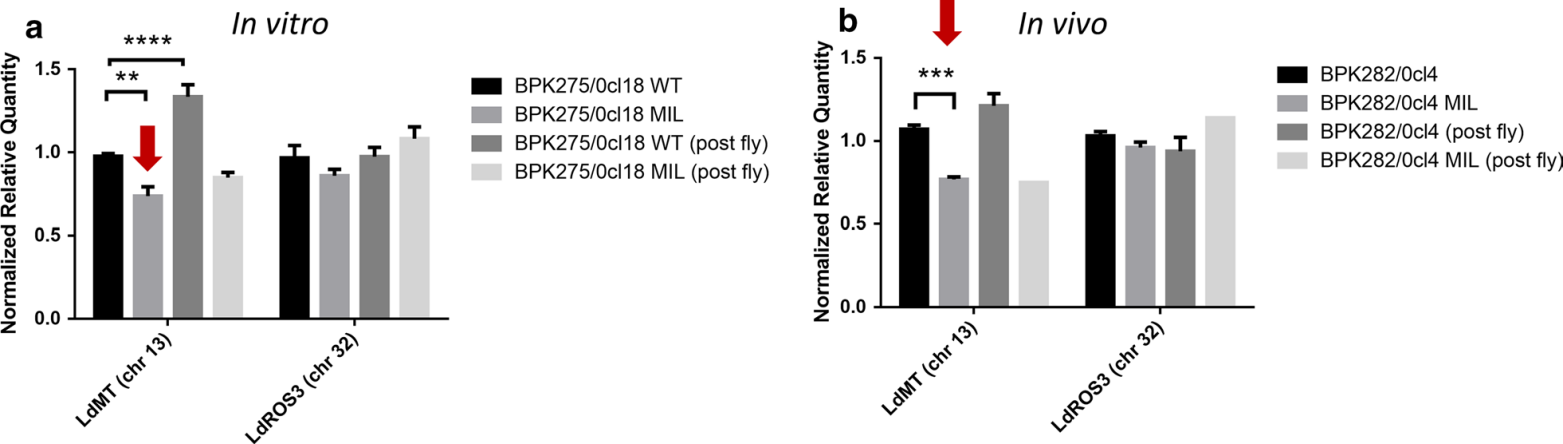
Comparison of the normalized relative quantity of *LdMT* (chromosome 13) and *LdROS3* (chromosome 32) for BPK275/0cl18 (**a**) and BPK282/0cl4 (**b**). A significant reduction in *LdMT* relative quantity could be detected between the WT and the *in vitro* and *in vivo* MIL-selected lines, and is compatible with WT being trisomic and the MIL-selected line disomic for chromosome 13 (arrows). A significant difference in *LdMT* could also be noted for the WT strain before and after passage in the sand fly. No significant differences could be detected in *LdROS3* gene copy number for the *in vivo*-selected. Results are based on duplicate measurements of at least three biological replicates and are expressed as the mean (± SE) relative quantity normalized using chromosome 24 (stably disomic), chromosome 31 (mostly tetrasomic) and chromosome 36 (stably disomic) as references. **P < 0.01, ***P < 0.001, ****P < 0.0001

**Fig. 2:**
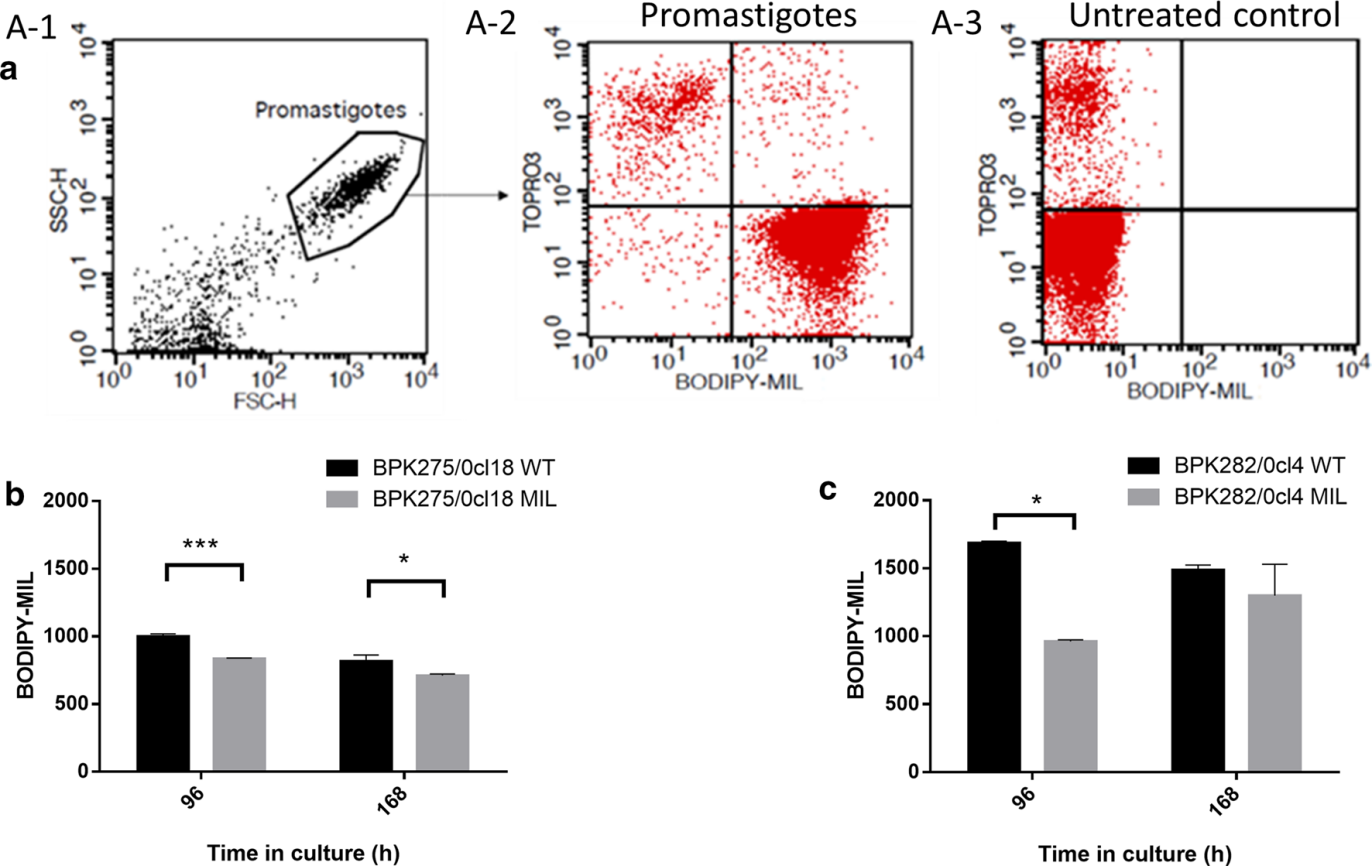
(**a**) Flow cytometric analysis of BODIPY-MIL-labelled promastigotes of BPK275/0-cl18 WT. A-1: Side scatter (SSC-H) *versus* forward scatter (FSC-H) dot plot with gated population corresponding to 0.5 µM MIL-treated promastigotes. A-2: Further characterization of promastigotes based on live/dead staining TO-PRO-3 and BODIPY-MIL fluorescence. A-3: Non-treated promastigotes (control sample). Excitation was set at 488 nm and emission was detected by FL2 channel. Flow cytometric analysis of BODIPY-MIL accumulation within 1 h exposure in log (96 h) and stationary-phase (168 h) promastigotes of the WT parent and *in vitro* (**b**) or *in vivo* (**c**) MIL-treated counterparts. Results are based on triplicate measurements of three biological replicates and are expressed as the mean FL2 fluorescence ± SE. *P < 0.05, ***P < 0.001

To check the involvement of aneuploidy, the relative abundance of the *LdMT* gene (chromosome 13) and the β-subunit gene *LdROS3* (chromosome 32) were compared between WT and MIL-selected parasites before and after passage in the sand fly. A decrease in *LdMT* copy number was noted after MIL selection for both strains (Fig. 1) and is compatible with a decrease in chromosome ploidy from trisomic to disomic state (BPK275: *F*_(3, 30)_ = 9.861, *P* = 0.0001; BPK282: *F*_(3, 16)_ = 13.12, *P* = 0.0001). BODIPY-MIL uptake studies demonstrated significantly decreased drug accumulation in log-phase promastigotes (96 h in culture) of both *in vitro* and *in vivo* MIL-selected parasite strains, and in stationary-phase (168 h in culture) of the *in vivo* MIL-selected strain (Fig. 2) (BPK275: *F*_(1, 4)_ = 4.155, *P* = 0.1111; BPK282: *F*_(1, 2)_ = 6.043, *P* = 0.1332). After sand fly passage, the *LdMT* gene copy number increased for the WT (Fig. 1), making it more susceptible to MIL than before passage through the vector (Table 1). The copy number did not significantly change in the MIL-selected strains isolated from sand flies.

### Parasite fitness analysis

#### Promastigote growth

**Fig. 3:**
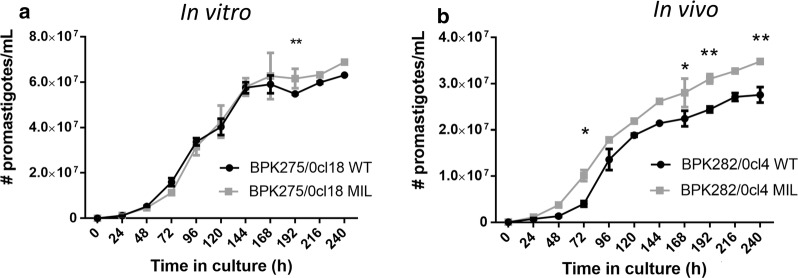
Promastigote growth curves of wild-type parent (WT) and derived counterparts that were repeatedly exposed to MIL, either *in vitro* (**a**) or *in vivo* (**b**). No biologically significant differences could be observed for the *in vitro* MIL-exposed strain (**a**) whereas successive selection cycles *in vivo* resulted in parasites with a significantly increased *in vitro* promastigote growth (**b**). Results are expressed as the mean number of parasites in culture ± SE and are based on three independent replicates. *P < 0.05, **P < 0.01

Promastigote multiplication revealed no biologically significant variations between the WT parent and the *in vitro* MIL-exposed counterpart (Fig. 3a). *In vivo* MIL exposure resulted in parasites with a significantly increased promastigote multiplication rate compared to its WT parent strain (Fig. 3b) (*F*_(10, 64)_ = 263.4, *P* < 0.0001).

#### Morphological evaluation of metacyclogenesis

**Fig. 4:**
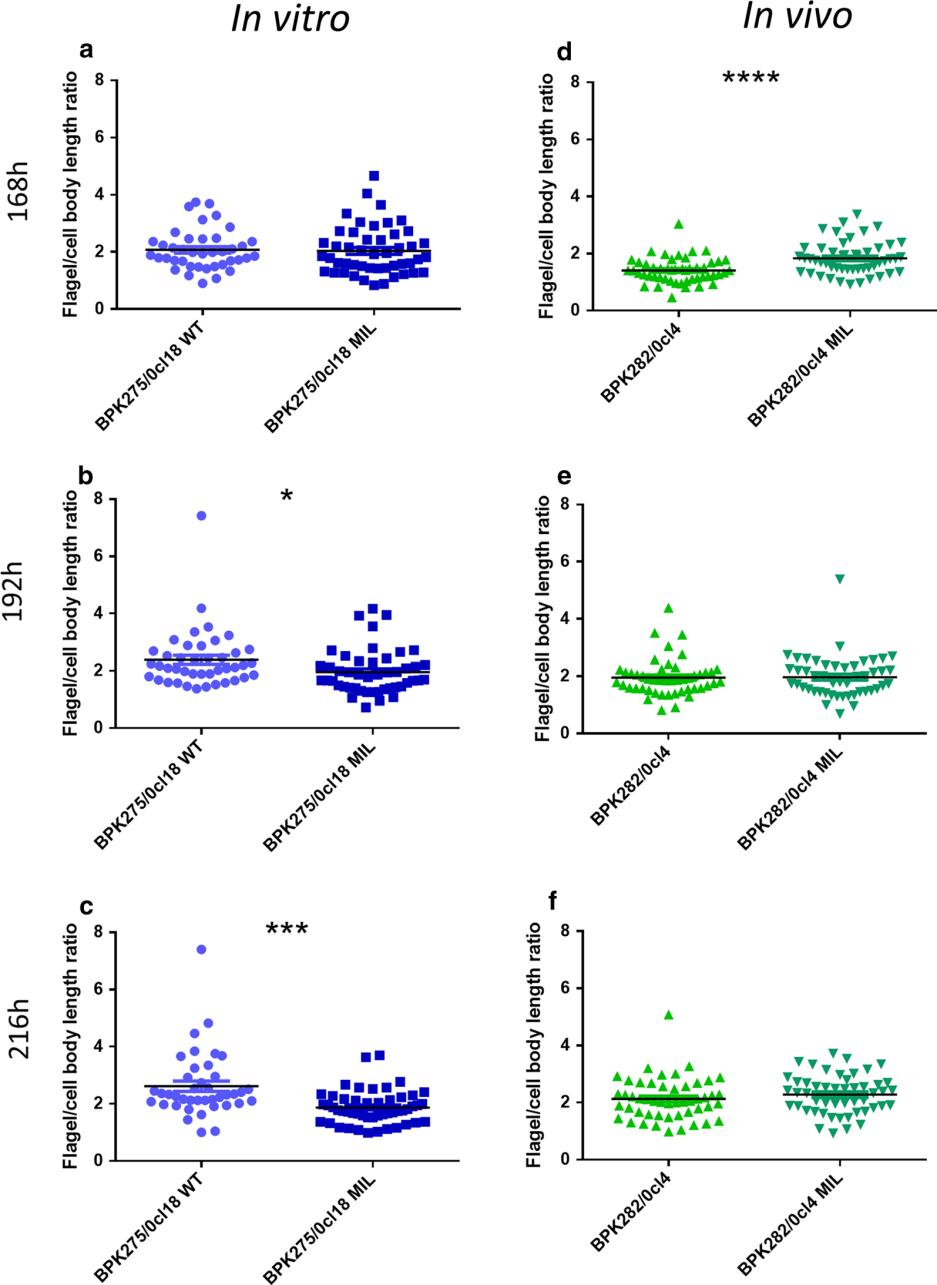
Morphological evaluation of metacyclogenesis of WT parent and derived counterparts that were repeatedly exposed to MIL either *in vitro* (**a**–**c**) or *in vivo* (**d**–**f**). Repeated exposure to MIL *in vitro* resulted in significantly lower flagellum/cell body length ratios in 192 h-old and 216 h-old cultures. However, the cut-off value for metacyclogenesis was reached in 168 h-old cultures for both the WT and MIL-exposed strain. Repeated MIL exposure *in vivo* resulted in significantly higher flagellum/cell body length ratios in 168 h-old cultures with cut-off values for metacyclogenesisonly being reached in 192 h-old cultures. Results are expressed as the mean ratio ± SE and are based on the measurement of at least 50 promastigotes. *P < 0.05, ***P < 0.001

Promastigote transformation into metacyclics (flagellum length/cell body ratio > 2) was affected after recurrent MIL treatment *in vitro*, whereas no impact of *in vivo* treatment could be demonstrated. Phenotypic differences upon *in vitro* selection were observed as an altered morphometric profile at the population level with a lower percentage (max 40%) of metacyclic promastigotes versus 80% in the WT population (Fig. 4) (BPK275: *F*_(5, 517)_ = 3.525, *P* = 0.0038; BPK282: *F*_(3, 388)_ = 0.4365, *P* = 0.7270).

#### Promastigote infectivity in macrophages

**Fig. 5:**
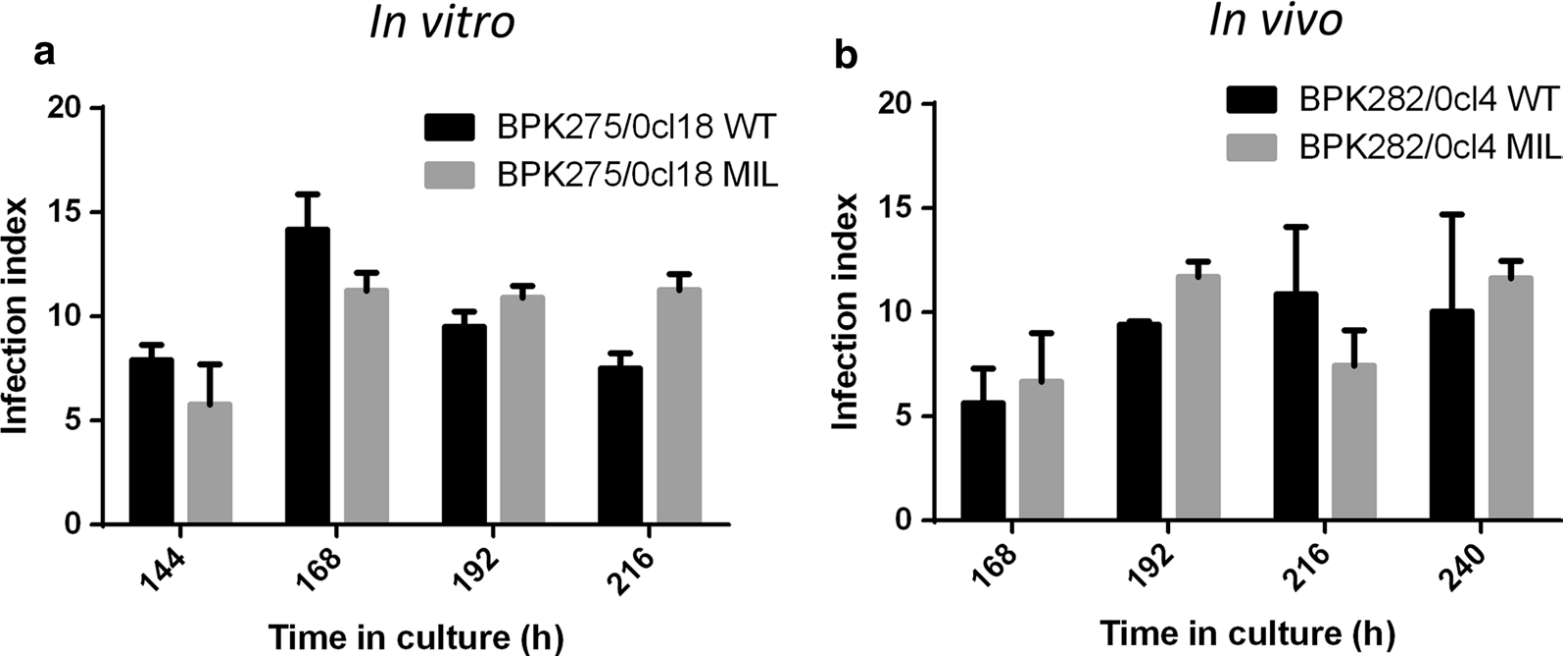
Promastigote infectivity of both WT parent and derived counterparts that were repeatedly exposed to MIL either *in vitro* (**a**) or *in vivo* (**b**). No significant differences in *in vitro* macrophage infectivity are observed. Maximum infectivity was reached with 168 h-old cultures for BPK275/0-cl18 and with 192 h/216 h-old cultures for BPK282/0-cl4. Results are expressed as the mean infection index ± SE and are based on three independent replicates

The highest infection ratios were reached when cells were infected with promastigotes from stationary-phase cultures in which metacyclics were most abundant. No effect was detected of both *in vitro* and *in vivo* MIL exposure on promastigote infectivity (Fig. 5) (BPK275: *F*_(3, 8)_ = 0.9287, *P* = 4701; BPK282: *F*_(4, 10)_ = 0.6246, *P* = 0.6556).

#### Promastigote multiplication and metacyclogenesis in the sand fly vector

**Fig. 6:**
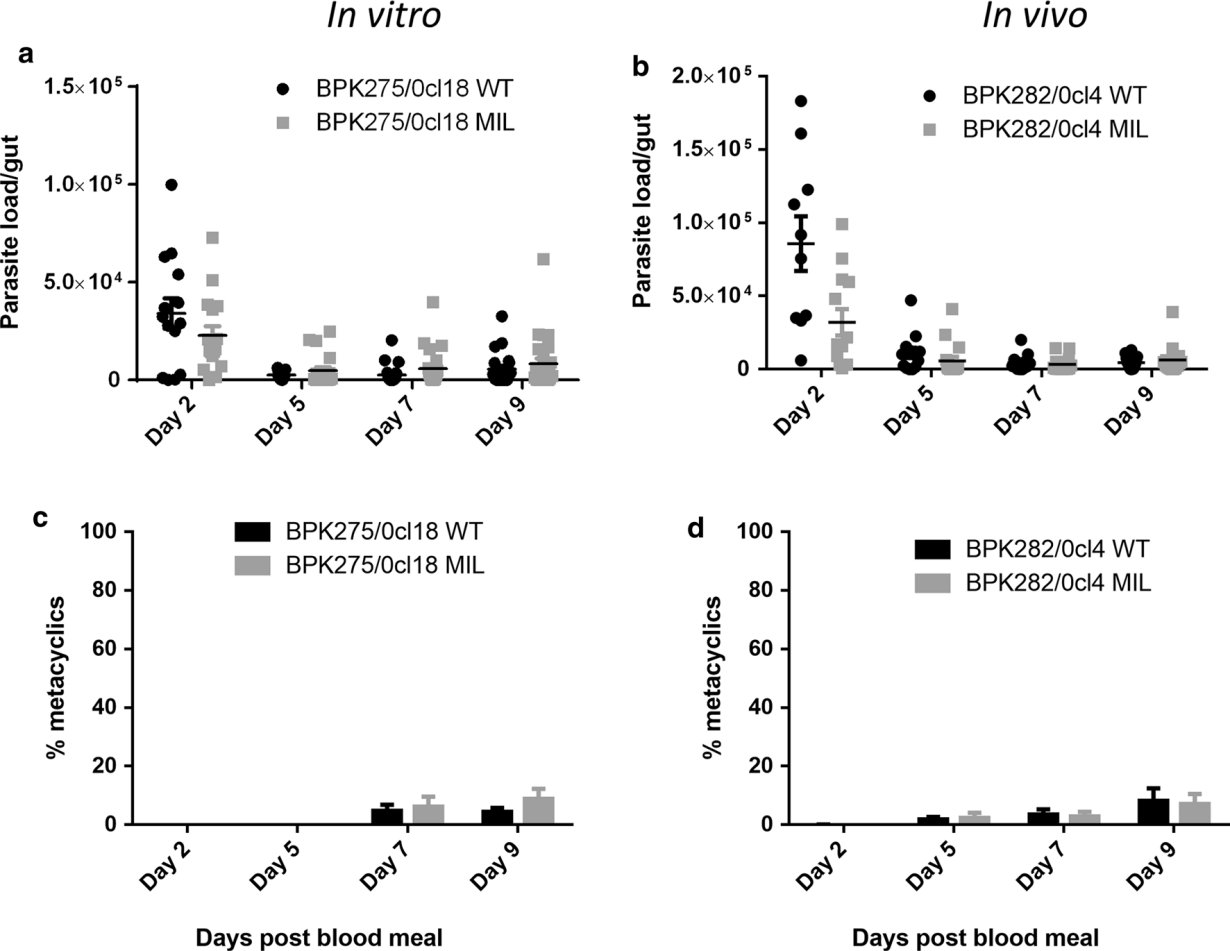
Total parasite load per gut (**a** and **b**) and the average percentage of metacyclic parasites (**c** and **d**) of WT- and MIL-exposed strains (MIL) in *L. longipalpis*. Sand fly infections were followed-up for 12 days after the infective blood meal. No significant differences could be observed between WT- and MIL-exposed strains. The represented data are combined from 2 independent experiments with a minimum of 10 flies for each group and time point

To compare the transmission potential, parasite survival and multiplication were evaluated in the *L. longipalpis* sand fly vector. When the total parasite load per gut and the percentage of metacyclic promastigotes were compared, no significant differences could be observed between the WT parent strains and the derived lines that were repeatedly exposed to MIL either *in vitro* (BPK275) or *in vivo* (BPK282) (Fig. 6) (BPK275: *F*_(8, 81)_ = 0.1893, *P* = 0.9918; BPK282: *F*_(3, 42)_ = 0.2239, *P* = 0.8793).

## Discussion

The growing number of MIL relapse cases in the Indian subcontinent is severely compromising the further use of MIL in monotherapy. Even though treatment failures have become fairly common, the exact causal reasons still remain to be elucidated. For example, involvement of host-related parameters linking treatment failure and post-treatment relapse to drug pharmacokinetics and dynamics (PK/PD) have already been indicated [26, 27] as well as the link of MIL relapse to both drug-related and parasite-related factors [28]. It was shown that a MIL-resistant phenotype can impact on parasite fitness [13, 14, 29] and that clinical MIL relapse may be associated with enhanced infectivity [3]. The present laboratory study specifically aimed to evaluate the phenotypic impact of repeated MIL exposure using syngeneic strains obtained through both *in vitro* and *in vivo* selection procedures [15, 16].

Given the sometimes difficult adaptation of clinical isolates to the hamster model, two reference strains from an EU-FP7 Programme (Kaladrug-R project 222895) were used in this study. Both were selected for their background susceptibility to antimony (Sb), with BPK275/0/-cl18 having a Sb-resistant background and BPK282/0-cl4 having a Sb-susceptible background. Although a Sb-resistance background may alter the interaction with several drugs [30], it does not impact on the experimental selection for MIL resistance [31], hence allowing the use of both strains without challenging our stated research aims. The intrinsically low susceptibility of BPK282/0-cl4 towards paromomycin, a feature that has been associated with increased infectivity, could possibly explain its relatively more easy adaptation to the hamster model [22].

Despite former reports on the rapid and straightforeward selection of MIL-resistance in promastigotes [31–33], successive selection cycles on intracellular amastigotes both *in vitro* and *in vivo* failed to result in a drug-resistant phenotype [15, 34], creating the possibility to assess the impact of repeated MIL exposure without the alterations in parasite fitness as observed with full MIL resistance [13, 14, 29]. In the present study, recurrent *in vitro* and *in vivo* MIL exposure only resulted in a marginal decrease in promastigote susceptibility, which corroborates other studies describing the existence of an ‘intermediate’ resistant phenotype characterized by a partial decrease in susceptibility as a step towards full resistance [31]. Post-treatment relapse in the field has been linked to only a minor decrease in MIL susceptibility, with this ‘intermediate’ resistant phenotype behaving similarly to the non-exposed wild-type strains [35]. This contrasts with other studies describing an increased infectivity for relapse-derived strains [3, 5] which is actually unexpected given the detrimental changes in fitness upon aqcuisition of full resistance [13, 14, 29].

A functional LdMT transporter has been shown to be imperative for intrinsic MIL susceptibility. In the ‘intermediate’ resistant phenotype, biochemical analyses revealed a moderate but significant reduction of MIL accumulation. Since efflux rates are not significantly increased (Additional file 1: Figure S1), outward drug transporters are likely not primarily involved in the observed decrease in MIL susceptibility. Similarly, clinical isolates with an increased tolerance to MIL have been reported to show a decreased drug uptake [5]. The lower steady-state MIL accumulation correlates with a lower *LdMT* gene copy number that probably arose from the parasite’s extreme genomic plasticity under stress conditions [36]. Specifically, the modified *LdMT* gene dosage could result from a decreased somy of chromosome 13 [31, 37]. Indeed, a qPCR-based analysis showed a significant decrease in normalized relative quantity of this chromosome for both strains after repeated MIL-exposure and is compatible with a change from a trisomic to disomic state.

As former research already demonstrated the possibility of emerging phenotypic heterogeneity [38, 39], the present study was carried out using the polyclonal population obtained after the repeated drug exposure. As a result of its genomic plasticity, the parasite can create phenotypic diversity by mosaic aneuploidy, i.e. varying chromosomal content between cells leading to intra-strain genomic heterogeneity [36]. The observed change from a trisomic to disomic state of chromosome 13 after repeated *in vitro* MIL exposure aligns with the ‘intermediate’ resistance on promastigote level. Corroborating a previous study, the moderately increased promastigote IC_50_ does not translate into susceptibility changes at the amastigote level [31]. Next to drug susceptibility, MIL-exposed parasites exhibited slightly altered growth and/or metacyclogenesis phenotypes under *in vitro* culture conditions. Despite these changes, parasite infectivity and metacyclogenesis remained unaltered in the sand fly vector: MIL-exposed lines appeared to retain a disomic state of chromosome 13 after sand fly passage. Recent research using polyclonal BPK275 demonstrated a swift adaptation to a disomic state of chromosome 13 upon passage in the sand fly [24]. This implies that the ‘intermediate’ resistant phenotype would be stable, as we indeed observed the disomic state of chromosome 13 to be conserved after sand fly passage. Nevertheless, absolute IC_50_ values seemed to be slightly lower following passage through the sand fly vector, but these could not be attributed to significant changes in *LdMT* or *LdRos3* gene dosage.

Collectively, our observations indicate that recurrent MIL exposure evokes a range of subtle phenotypic changes, without significant impact on sand fly infection and development of infective parasite stages.

## Conclusions

Repeated *in vitro* and *in vivo* exposure of *Leishmania* parasites to MIL evokes a number of subtle phenotypic changes in promastigotes, including a slightly reduced MIL susceptibility. This was found to be associated with a reduced *LdMT* gene copy number and reduced drug accumulation. Despite these phenotypic changes, parasite infectivity and metacyclogenesis remained unaltered inside the sand fly vector.

## Supplementary information

**Additional file 1: Text S1.** Methods. **Table S1.** Primer sequences and qPCR settings used to evaluate the MIL transporter copy number by qPCR. **Text S2.** Results. **Figure S1.** Time-dependent efflux of BODIPY-MIL. Delta FL2/min was calculated for log-phase (96 h) and stationary-phase (168 h) promastigotes of WT and MIL-exposed strains. Efflux rates were not significantly altered. Results are based on triplicate measurements of three biological replicates and are expressed as the average of delta FL2/min ± SE.

## Data Availability

All data generated or analyzed during this study are included in this published article. The raw datasets are available from the corresponding author upon reasonable request.

## Abbreviations

dpi: Days post-infection
IC_50_: Inhibitory concentration 50%
LdMT: *Leishmania donovani* miltefosine transporter
MIL: Miltefosine
PBS: Phosphate-buffered saline
Sb: Antimony
SE: Standard error of the mean
s.i.d: Single injection dose
VL: Visceral leishmaniasis
WT: Wild-type
