# Supplementary material for: Phenotypic adaptations of Leishmania donovani to recurrent miltefosine exposure and impact on sand fly infection
Source: Parasit Vectors. 2020 Feb 22;13:96. doi: 10.1186/s13071-020-3972-z (PMC7036194; doi:10.1186/s13071-020-3972-z)

**ADDITIONAL FILE 1**

**Phenotypic adaptations of *Leishmania donovani* to recurrent miltefosine exposure and impact on sand fly infection**

S Hendrickx^1^, L Van Bockstal^1^, D Bulté^1^, A Mondelaers^1^, H. Aslan^1^, L Rivas^2^, L Maes^1^, G Caljon^1#^

^1^ Laboratory of Microbiology, Parasitology and Hygiene (LMPH), University of Antwerp, Antwerp, Belgium

^2^ Centro de investigaciones Biológicas– CSIC, Madrid, Spain

# Text S1. Methods

## *MIL transporter copy number comparison by qPCR*

Given the known plasticity in chromosome copy number for chromosome 13 (harboring the *LdMT* MIL-transporter gene) and chromosome 32 (harboring the *LdROS3*-subunit gene), the copy number of both was determined by qPCR. The Step One Plus real-time PCR system (Applied Biosystems, Belgium) was used for all PCR assays and melt curve analyses. Each assay was run in technical duplicate according to the conditions stated in Table S1

**Table S1: Primer sequences and qPCR settings used to evaluate the MIL transporter copy number by qPCR.**

| **Targets** | **Primer sequences** | **Activation step** | **Amplification** | **Melting curve** |
| --- | --- | --- | --- | --- |
|  |  |  | **(40 cycles)** |  |
| ***LdMT (XP_001464173.1)*** | | | | |
| FP | 5’-AGTCAACCGGTAAGACAGCG-3' | 95°C 10min | 95°C 30 s, 60°C 1min | 95°C 15s, 60°C 1min, 95°C 15s (step & hold with 0.3°C increments) |
| RP | 5'-GTGCACCTTCCAGCCCAATA-3' |  |  |  |
| **Chromosome24 *(XP_003861104.1)*** | |  |  |  |
| FP | 5’- GGCTCAGTAACGTGCAGAAG-3’  5'-GTTGTCGAGGAGGCTGAAGA-3’ | 95°C 10min | 95°C 30 s, 60°C 1min | 95°C 15s, 60°C 1min, 95°C 15s (step & hold with 0.3°C increments) |
| RP |  |  |  |  |
| **Chromosome 31 *(XP_003863199.1)*** | |  |  |  |
| FP | 5’-GGCCAACCACCATGCCTCAA-3’ | 95°C 10min | 95°C 30 s, 60°C 1min | 95°C 15s, 60°C 1min, 95°C 15s (step & hold with 0.3°C increments) |
| RP | 5’-ACGCAAGACCGCAGATGAGC-3’ |  |  |  |
| ***LdROS3 (XP_001467750)*** | |  |  |  |
| FP | 5’-AAAACCGCATTGAGCAGCAG-3’ | 95°C 5min | 95°C 15 s, 60°C 30s | 95°C 15s, 60°C 1min, 95°C 15s (step & hold with 0.3°C increments) |
| RP | 5’-CCCGCTCACAATTACGAGGA-3’ |  |  |  |
| **Chromosome 36 *(XP_003865139.1)*** | |  |  |  |
| FP | 5’-GAATGGCGACGACGAGGACT-3’ | 95°C 5min | 95°C 15 s, 60°C 30s | 95°C 15s, 60°C 1min, 95°C 15s (step & hold with 0.3°C increments) |
| RP | 5’-TTGGAGCGGACGGTGTTACG-3’ |  |  |  |

## *Evaluation of MIL efflux rates*

After measurement of steady-state BODIPY-MIL uptake, the same set of samples was placed at 25°C after which the decline of fluorescence intensity was analyzed in live cells at different time points (60 minutes, 120 minutes and 180 minutes) to assess MIL efflux, the average decrease in BODIPY-MIL fluorescence per minute were obtained every hour after the start of the efflux experiment (intervals: 0-60min; 60-120min; 120-180min). Results are based on triplicate measurements of three biological replicates.

***Statistical analysis***

All statistical analyses were performed using Graphpad Prism version 6.00 software. Statistical differences between the MIL-uptake and efflux rates of WT parent and MIL-exposed derived strains and between the different time points within one group were determined using an unpaired Student’s *t*-test. Tests were considered statistically significant if *p* <0.05.

# Text S2. Results

To evaluate whether the differences in steady-state drug accumulation could result from an altered efflux transport of BODIPY-MIL, labelled parasites were incubated at 25°C and the decline of fluorescence intensity was measured for up to 180 minutes. Calculation of the MIL efflux (delta FL2/min) revealed no significantly increased efflux in MIL-treated versus WT strains at each selected time point showing that the lower drug accumulation in the *in vitro* MIL-selected line may not be due to an increased efflux, but was rather caused by a lower rate of drug uptake (Figure 2)

Figure S1: Time-dependent efflux of BODIPY-MIL. Delta FL2/min was calculated for log-phase (96h) and stationary-phase (168h) promastigotes of WT and MIL-exposed strains. Efflux rates were not significantly altered. Results are based on triplicate measurements of three biological replicates and are expressed as the average of delta FL2/min ± SEM.


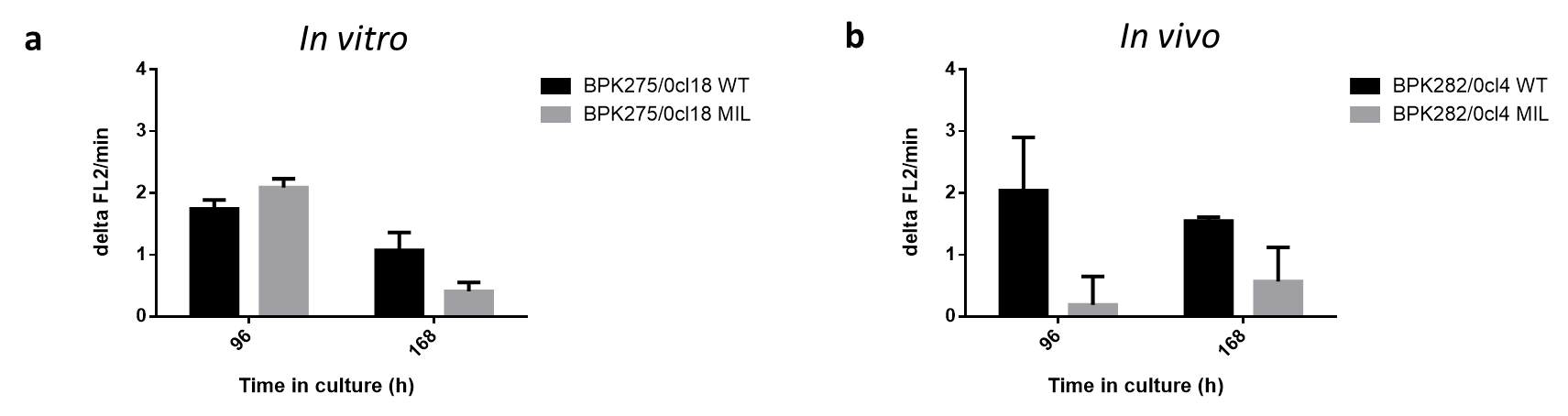

Supplement: Supplementary file 1 — Additional file 1: Text S1. Methods. Table S1. Primer sequences and qPCR settings used to evaluate the MIL transporter copy number by qPCR. Text S2. Results. Figure S1. Time-dependent efflux of BODIPY-MIL. Delta FL2/min was calculated for log-phase (96 h) and stationary-phase (168 h) promastigotes of WT and MIL-exposed strains. Efflux rates were not significantly altered. Results are based on triplicate measurements of three biological replicates and are expressed as the average of delta FL2/min ± SE. [file 13071_2020_3972_MOESM1_ESM.docx]
